# On a New Method of Using Needles in the Operation for Harelip

**Published:** 1870-01

**Authors:** L. Tait


### On a New Method of Using Needles in the Operation for Harelip.

I venture to bring before the notice of practical suigeons an improvemtnt in this operation, which I believe to be of importance. It is an operation which is performed principally for æsthetic reasons, and it is frequently marred in this respect by the ungainly scars which are left by the needles when used in the ordinary way; in fact, I have not yet seen a case in which this defect has not been marked. What I propose is, that, instead of two or more needles being introduced transversely through the flaps, they should be used in this manner: Having made what incisions he deems requisite for the operation (and I may here say that I have abandoned all the fancy manipulations for the old-fashioned straight incisors, removing plenty of tissue), the surgeon is to introduce two ordinary seamstress's needles, armed with a few inches of silver wire doubled, through the flaps, in the form of a St. Andrew's cross; the point of each needle is to be introduced through the mucous membrane of the lip, about half an inch from the edge of the flap, and brought out at the middle of the incision, then introduced into the other flap at the point opposite, and brought out at the root of the ala of the nose. The needles cross in the middle of the wound. The flaps are to be carefully adjusted, then the heads of the needles to be pushed fairly into the lip, and pulled together by twisting the wires; the points of the needles are then to be cut off close to the skin, and the stumps retracted into the flesh. In this way nothing is left to "catch," and, when the needles are removed, by untwisting the wires and pulling by them, there are no scars left.

In the last case in which I used this method, the parents of the child, aged seven, say that it is scarcely possible for a stranger to tell that the child had been operated upon, and in this case there was a complete and very wide intermaxillary cleft, which I had previously closed.—L. Tait, in *Lancet—Leavenworth Med. Herald.*

